# Clinical Utility of Induced Sputum and Bronchoalveolar Lavage Cultures in Diagnosing Nontuberculous Mycobacterial Pulmonary Disease

**DOI:** 10.3390/pathogens13121064

**Published:** 2024-12-03

**Authors:** Maria Angela Licata, Paola Mencarini, Annelisa Mastrobattista, Serena Maria Carli, Carlotta Cerva, Silvia Mosti, Raffaella Libertone, Alberto Zolezzi, Pietro Vittozzi, Carla Nisii, Antonio Mazzarelli, Angela Cannas, Assunta Navarra, Stefania Ianniello, Rocco Trisolini, Delia Goletti, Fabrizio Palmieri, Gina Gualano

**Affiliations:** 1 Respiratory Infectious Diseases Unit, National Institute for Infectious Diseases “Lazzaro Spallanzani” IRCCS, 00149 Rome, Italy; maria.licata@inmi.it (M.A.L.); a.mastrobattista@inmi.it (A.M.); serena.carli@inmi.it (S.M.C.); carlotta.cerva@inmi.it (C.C.); silvia.mosti@inmi.it (S.M.); raffaella.libertone@inmi.it (R.L.); alberto.zolezzi@inmi.it (A.Z.); pietro.vittozzi@inmi.it (P.V.); fabrizio.palmieri@inmi.it (F.P.); 2 Department of Microbiology, National Institute for Infectious Diseases “Lazzaro Spallanzani” IRCCS, 00149 Rome, Italy; carla.nisii@inmi.it (C.N.); antonio.mazzarelli@inmi.it (A.M.); angela.cannas@inmi.it (A.C.); 3 Department of Epidemiology, National Institute for Infectious Diseases “Lazzaro Spallanzani” IRCCS, 00149 Rome, Italy; assunta.navarra@inmi.it; 4 Diagnostic Imaging Unit for Infectious Diseases, National Institute for Infectious Diseases “Lazzaro Spallanzani” IRCCS, 00149 Rome, Italy; stefania.ianniello@inmi.it; 5 Interventional Pulmonology Division, Fondazione Policlinico Universitario “Agostino Gemelli” IRCCS, 00136 Rome, Italy; rocco.trisolini@policlinicogemelli.it; 6 Translational Research Unit, National Institute for Infectious Diseases (INMI), “Lazzaro Spallanzani” IRCCS, 00149 Rome, Italy; delia.goletti@inmi.it

**Keywords:** nontuberculous mycobacterial pulmonary disease (NTM-PD), non-tuberculous mycobacteria (NTM) culture, real-time polymerase chain reaction (PCR), induced sputum (IS), bronchoalveolar lavage (BAL)

## Abstract

Diagnosing non-tuberculous mycobacterial pulmonary disease (NTM-PD) in patients unable to produce sputum spontaneously requires invasive procedures to obtain valid respiratory specimens. In this retrospective study, we evaluated the results of microbiological tests performed on respiratory samples of 132 patients affected by NTM-PD. In the diagnostic workout, 98 patients performed both induced sputum (IS) and bronchoalveolar lavage (BAL) and were enrolled in our study. A total of 93 out of 98 BAL samples (95%) were culture-positive for mycobacteria, whereas only 67/153 (44%) induced sputum cultures were positive for NTM (*p* < 0.001). Molecular identification of NTM with real-time polymerase chain reaction (PCR) was positive in 48/64 BAL (75%) and in 47/139 (34%) IS samples (*p* < 0.001). Patients affected by nodular-bronchiectatic form were 65/98 (66%): BAL culture was positive in 95% of cases (62/65 BAL), while only 30/99 IS cultures were positive (30%; *p* < 0.001). PCR was positive in 76% of BAL samples examined (26/34) and in 26% of the IS samples (24 out of 91) (*p* < 0.001). Among 33 patients with a fibro-cavitary radiological pattern, 65% of IS (35/54) were culture-positive for NTM, whereas 94% of cases (31/33) had a positive culture for NTM from BAL (*p* = 0.002). PCR was positive in 73% of BAL samples tested (22/30) and 48% of IS samples tested (23/48) (*p* = 0.031). Our results confirm BAL mycobacterial culture as the gold standard for the diagnosis of pulmonary mycobacteriosis. FBS with BAL should be performed in every patient with a strong suspicion of NTM-PD, if other respiratory samples are repeatedly negative. Sputum induction is a useful technique to obtain valid respiratory samples when patients are unable to produce spontaneous sputum, especially in the outpatient setting. However, during the diagnostic workup of NTM-PD, we should not forget that PCR and mycobacterial culture of induced sputum have a lower yield than when performed on BAL, especially in the nodular-bronchiectatic form of the disease.

## 1. Introduction

“Nontuberculous mycobacteria” (NTM) is a term that groups more than 190 species of mycobacteria, only some of which cause disease in humans [1]. Pulmonary localization of nontuberculous mycobacteria causes disease in predisposed individuals, with an insidious onset and nonspecific respiratory symptoms [1].

Current guidelines for the diagnosis of nontuberculous mycobacterial lung disease (NTM-LD or PD) require that clinical, radiological, and microbiological criteria be met [2,3]. The microbiological criterion requires positive culture results on at least two separate sputum specimens or from a single bronchial lavage or washings [2]. The American Thoracic Society/Infectious Diseases Society of America (ATS/IDSA) guidelines recommend collecting three sputum samples on different days [3], while the British Thoracic Society (BTS) guidelines require at least two sputum samples collected on separate days in every suspected case of pulmonary mycobacteriosis [4,5,6,7].

Spontaneous sputum (SS) and induced sputum (IS) are respiratory samples produced by patients with cough. They originate from the entire respiratory tract and do not originate exclusively from lung lesions. Sputum induction is an effective way to obtain a valid respiratory sample, already validated for the diagnosis of tuberculosis [8,9], which is safe, not expensive, and non-invasive.

With fibrobronchoscopy (FBS), bronchoalveolar lavage (BAL) is obtained by selective aspiration and saline washing of the lower respiratory tract, and it can be directed to specific parts of the lungs based on chest computed tomography. BAL facilitates the diagnosis of NTM-PD, since many patients, more often elderly or those affected by nodular non-excavated forms, are not able to produce valid respiratory samples for microbiological examination [2,7,10]. In clinical practice, spontaneous sputum, IS, and BAL are requested in sequence, because if one fails, the other may allow the diagnosis, as occurs for tuberculosis.

Several studies have reported that BAL cultures are more sensitive than sputum culture for the diagnosis of NTM-PD [11,12,13,14], but they have not focused on the diagnostic yield of IS. Finally, to date, no study has systematically compared the efficacy of molecular biology techniques and mycobacterial culture on IS and BAL in the diagnosis of pulmonary mycobacteriosis. This study therefore aims to evaluate the diagnostic yield of IS and BAL in NTM-PD in daily clinical practice.

## 2. Materials and Methods

### 2.1. Study Design

**Figure 1:**
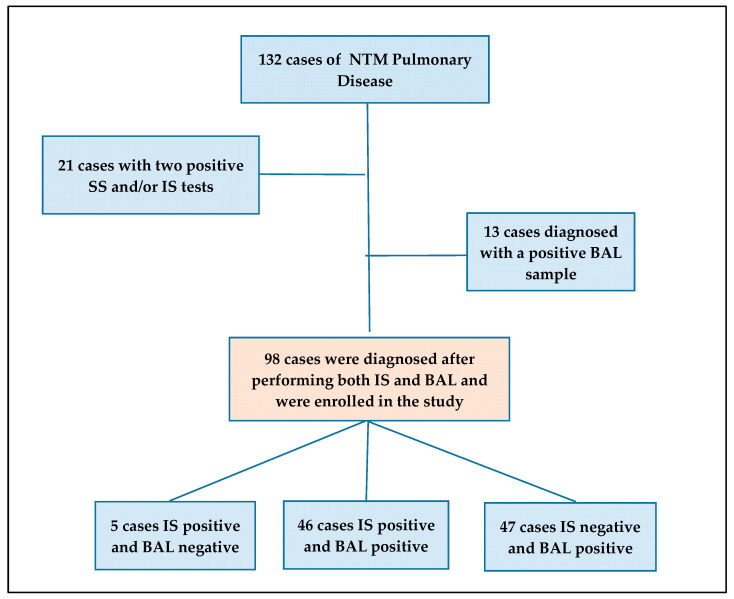
Flowchart: cases enrolled in the study according to the diagnostic methods. (ABBREVIATIONS: NTM: nontuberculous mycobacteria; SS: spontaneous sputum; IS: induced sputum; BAL: bronchoalveolar lavage).

This retrospective observational study was conducted at the Respiratory Infectious Diseases Unit, National Institute for Infectious Diseases (INMI) “L. Spallanzani” Scientific Research and Treatment Institute (IRCCS), Rome, Italy, a tertiary care center for mycobacterial diseases. We examined 132 patients with NTM-PD observed from May 2018 to December 2023. All patients met the criteria for the diagnosis of NTM-PD [3,4]. For the study, we eliminated 21 patients who had two culture-positives of SS, without having performed BAL, and 13 patients in whom the diagnosis was made by BAL alone, without examining sputum samples. We then considered a sample of 98 patients, of whom we examined at least one induced sputum sample and BAL (Figure 1). The study was approved by the INMI Ethics Committee, “L. Spallanzani”, with decision no. 12 (17 February 2015); all enrolled patients provided written informed consent to the use of anonymized clinical data. We collected demographic, clinical, and laboratory data from medical records: age, sex, NTM species, PCR and culture results for NTM on BAL and IS, radiological pattern, and presence of comorbidities and/or immunosuppression.

### 2.2. Procedures

During sputum induction, hypertonic saline solution (saline concentration 2–3%) was delivered to the patient with an ultrasonic nebulizer at high flows at 5 min intervals for a total of 20 min [15]. Then, the operator macroscopically assessed the validity of the sample before sending it to the microbiology laboratory. Bronchoscopy and bronchoalveolar lavage (BAL) were performed for diagnostic purposes, directing the bronchoscope into the affected lung segments and collecting samples after the instillation of three or five 20 mL aliquots of sterile saline (0.9% NaCl) [16]. BAL samples (minimum 15 mL) were promptly sent to the Microbiology laboratory and analyzed within two hours of collection.

Microbiological analysis included a smear examination and culture in a Mycobacteria Growth Indicator Tube (MGIT) and in a Lowenstein–Jensen (LJ) medium. Molecular identification of mycobacteria in primary samples or culture isolates was achieved by real-time PCR (Anyplex MTB/NTM Real-time Detection kit, Seegene, Seoul, Republic of Korea), and species identification and resistance were evaluated using the GenoType NTM-DR assay (Hain Lifescience, Nehren, Germany). This is a line probe assay (LPA) that enables species- or subspecies-level identification of the major clinically encountered NTM, including *Mycobacterium avium* complex (MAC) species (*M. avium*, *M. intracellulare*, and *M. chimaera*); *M. chelonae*; and subspecies belonging to *M. abscessus*, i.e., *M. abscessus* subsp. *abscessus*, *M. abscessus* subsp. *massiliense*, and *M. abscessus* subsp. *bolletii*. The NTM-DR assay also allows the detection of antibiotic resistance to macrolides and aminoglycosides in isolates from cultures. Briefly, nucleic acids were isolated from the specimen, amplified, and detected via a hybridization and alkaline phosphatase reaction on a membrane strip, according to the manufacturer’s instructions.

### 2.3. Statistical Analysis

Descriptive analysis was conducted to characterize subjects enrolled in the study. Categorical variables were expressed as numbers and percentages. Age (in years) was reported as continuous variable and was expressed as median and interquartile range (IQR). Baseline clinical and demographic data were compared between two independent groups using the chi-squared test, Fisher’s exact test, or Mann–Whitney test, as appropriate. The proportions of NTM detected by IS and BAL were compared using the two-proportion test. All statistical tests were two sided, and *p*-values < 0.05 were considered significant. Statistical analyses were conducted using MEDCalc (https://www.medcalc.org/calc/, accessed on 3 October 2024).

## 3. Results

- **Clinical-demographic data**

Characteristics of the 98 NTM-PD patients who underwent both IS and BAL are shown in Table 1. The median age was 68 years (IQR 62–76.25). Of the 98 patients in the study, 35 were men and 63 women (*p* = 0.005), without a clear association with comorbidities, immunosuppression, and age. We can hypothesize that we probably selected patients with difficulty in spontaneous expectorating (Figure 1), who could correspond at least in part to the description of Lady Windermere syndrome (recurrent in the literature), with a selective pressure in favor of women in our group.

New cases of NTM-PD accounted for 85% (83/98). Patients with comorbidities comprised 91% (89/98) of the sample. Total comorbidities were 168 (168/98; 1.7 per patient). The most frequent comorbidities were COPD/bronchiectasis (52/98; 53%); hypertension and other cardiovascular conditions (42/98; 43%); neoplasms (19/98; 19%); GERD (18/98; 18%); liver disease (8); diabetes (6); renal failure (5); autoimmune disease (3); and hypothyroidism (3). Seventeen patients were classified as immunosuppressed due to the comorbidities and the therapies they were undergoing (Table 1 and Table 2).

Patients with a nodular bronchiectatic radiological pattern represented 66% of the sample, with a female predominance (75%), while those with a fibro-cavitary radiological pattern represented 34%, with no significant gender difference (Table 2).

- **Microbiological data**

The culture results for NTM species were as follows: *M. avium* 32% (31/98), *M. chimaera* 30% (29/98), *M. intracellulare* 21% (21/98), *M. abscessus* spp. 5% (5/98), *M. kansasii* 5% (5/98), *M. xenopi* 4% (4/98), *M. fortuitum* 2% (2/98), and *M. scrofulaceum* 1% (1/98) (Table 1). Among the patients, 83% (81/98) were culture-positive for *Mycobacterium avium* complex (MAC): *M. avium*, *M*. *intracellulare*, and *M. chimaera* (Table 1 and Table 2). Compared to *M. avium* and *M. intracellulare*, the infections from *M. chimaera* seemed to be more frequently recurrent (10 recurrence vs. 19 new cases compared to 5 recurrences vs. 47 new cases (*p* = 0.006), showing a statistically significant difference (Table 1).

- **Diagnostic yield of IS and BAL mycobacterial culture**

During the diagnostic workup, NTM culture of induced sputum was positive in 67 out of 153 samples (44%) (Table 3). In the same period, all 98 patients underwent BAL, whose cultures were positive in 93 out of 98 patients (95%). BAL culture has shown a higher diagnostic yield than IS (*p* < 0.001) (Table 3 and Table 4).

- **Diagnostic yield of IS and BAL NTM molecular detection**

Respiratory samples were also examined by PCR for NTM in real time: 47 in 139 IS samples (34%) and 48 in 64 BAL samples (75%) were positive (Table 3). The diagnostic yield of PCR on BAL was confirmed to be better than on induced sputum also in this case (*p* < 0.001).

- **Correlation of IS and BAL microbiological findings with radiological appearance of lung lesions**

We divided the 98 patients into two groups according to the type of radiologically confirmed lung lesions: 65 (66%) had nodular bronchiectatic form while 33 (34%) had a fibro-cavitary form (Table 2). In Table 5, results of microbiological examinations are arranged following the radiological presentation of patients.

Only 30% of IS (30/99 samples) from patients with a nodular bronchiectatic radiological pattern was culture positive, while 95% (62/65) had a positive culture for NTM on BAL (*p* < 0.001). Among patients with fibro-cavitary radiological patterns, 65% of the IS examined (35/54) was culture-positive for NTM, while 94% of BAL (31/33) was culture positive (*p* = 0.002) (Table 4). The data show that the diagnostic yield of IS culture was higher in the fibro-cavitary pattern than in the nodular bronchiectatic pattern (65% versus 30%; *p* < 0.001). BAL culture was positive for NTM in 94% of patients with a fibro-cavitary pattern and in 95% of those with a nodular bronchiectatic pattern, without a statistically significant difference (*p* = 0.836) (Table 4).

The analysis of PCR results on BAL and IS is reported in Table 6. Twenty-four of the 91 IS from nodular bronchiectasis lung disease (26%) were positive by PCR, while we found 26 positive BAL out of the 34 tested by PCR (76%), with a statistically positive difference between the two types of respiratory specimens (*p* < 0.001). In fibrocavitary forms, 23/48 (48%) IS samples were PCR positive, and 22/30 (73%) of the tested BAL were positive, with a significant difference (*p* = 0.031) (Table 6).

Our data show that the diagnostic yield of IS PCR was higher in the fibro-cavitary pattern than in the nodular bronchiectatic pattern (48% versus 26%; *p* = 0.009). BAL PCR was positive for NTM in 76% of patients with nodular bronchiectatic pattern and in 73% of those with a fibro-cavitary pattern, without a statistically significant difference (*p* = 0.785) (Table 6).

- **Comparison between culture and PCR**

We compared the efficacy of culture and PCR in detecting NTM in induced sputum and BAL samples and found that induced sputum culture was positive in 67/153 (44%) compared to 47/139 (34%) positive with PCR (*p* = 0.0810). With BAL, this difference was statistically significant: 93/98 (95%) positive by culture compared to 48/64 (75%) positive by PCR, *p* < 0.001 (95%CI 9.1% to 32.2%) (Table 7).

## 4. Discussion

The incidence of NTM lung disease is constantly increasing and constitutes a source of growing concern for public health, as the disease overall affects immunosuppressed people, people affected by pre-existing lung-predisposing disease and conditions, or elderly subjects suffering from different comorbidities [17]. Diagnosis and treatment of NTM-PD are challenging for several reasons [18]. Identification of mycobacteria and their subtypes is crucial for the choice of a specific treatment [19], but not all microbiology laboratories can cultivate and differentiate atypical mycobacteria, and not all healthcare facilities have clinical experience and skills to follow patients affected by NTM during the treatment period.

The application of the diagnostic criteria for NTM-PD according to the current guidelines, proposed by the American Thoracic Society (ATS), the Infectious Diseases Society of America (IDSA), and British Thoracic Society (BTS), include clinical, radiological, and microbiological components [2,3,4]. In the microbiological criterion, spontaneous sputum, induced sputum, bronchial washings, bronchoalveolar lavage specimens, and transbronchial biopsies are all mentioned for the diagnosis of NTM lung disease.

Certainly, these criteria aid the clinician, but the particularities of some NTM species, the variability of radiological patterns, and the difficulty in obtaining valid respiratory samples in some patients sometimes make the application of the microbiological criterion difficult, with consequent possible delay in diagnosis.

In the 98 NTM-PD patients evaluated in this study, who underwent a diagnostic workup that included both IS and BAL, the diagnostic yield of mycobacterial culture was 44% for IS and 95% for BAL (*p* < 0.001). This difference was statistically significant when comparing the diagnostic yield of IS and BAL culture in patients with fibro-cavitary form (*p* < 0.05) and even more in patients with nodular bronchiectatic form (*p* < 0.001).

Holt and colleagues (2020) [20] compared the diagnostic yield of spontaneous sputum with induced sputum in 93 unselected patients suspected of NTM-PD. They found concordance of culture results between spontaneous sputum and induced sputum (81% versus 78%; *p* = 0.86).

Sputum induction is a non-invasive, cost-effective procedure suitable for the outpatient clinical setting, which in many cases solves the need to obtain an adequate respiratory sample for cytological and microbiological examinations [8,9,15].

However, discordant data on the diagnostic power of IS and BAL for NTM-PD diagnosis are reported in the literature [2,3]. Several studies have highlighted the high diagnostic yield of bronchoscopy for NTM-PD, ranging from 57.8% to 93.8% [10,12]. In some small studies, BAL fluid cultures have been reported to be more sensitive than spontaneously expectorated sputum cultures to diagnose nodular bronchiectatic NTM disease [11,13,14]. However, in another study, Ikedo Y. concluded that the yield of sputum culture and BAL culture was equivalent [19]. In the meta-analysis by Luo et al. [9], it is stated that in smear-negative tuberculosis patients, sputum induction and FBS show similar diagnostic power. However, the diagnostic yield of a sputum specimen for tuberculosis, which requires only a positive culture, does not automatically apply to NTM-PD, as the diagnosis of NTM-PD requires at least two positive sputum cultures together with the assessment of clinical and radiological criteria [2,3].

Real-time PCR testing for NTM-DNA was positive in 34% of IS samples and 75% of BAL samples (*p* < 0.001), with a lower performance than culture, especially in BAL samples, but its advantage is that results are available rapidly. It allows rapid confirmation in highly suspected cases, allowing treatment to be started pending culture and species identification.

In his retrospective study, Michael R. Holt stated that performing cultures of three induced sputum samples collected on separate days can increase the diagnostic power of the method in patients with NTM-PD, resulting in a culture positivity on IS of 78% [20]. Urabe et al. [6] retrospectively reported the increase in culture positivity of 16.5% with the third spontaneous sputum, allowing the diagnosis of NTM-PD in 23/139 patients. Furthermore, the most relevant guidelines [2,3,4] support the previous statement that sputum culture is usually sufficient for diagnosis in patients with NTM-PD in its fibro-cavitary form, if they can expectorate.

However, in our study, BAL culture was essential in making the diagnosis of NTM-PD in 47/98 patients: in 38 out of 65 cases (58%) with nodular bronchiectatic presentation and in 9 out of 33 cases (27%) with fibro-cavitary form (Table 5).

Bronchoscopy is a relatively safe method with rare complications. Since its introduction in 1960, published complication rates range from 0.1% to 11%, with mortality generally between 0 and 0.1% [21,22]. BAL is a minimally invasive technique with a low complication rate (0–2.3%) and no associated mortality. Major complications can occur in patients with severe lungs or heart disease [16].

In centers where it is possible to use a bronchoscopy-dedicated room with adequately trained staff, BAL is a safe and effective diagnostic tool, even if it has higher costs than spontaneous or induced sputum.

This study has several limitations, including its retrospective design; small sample size; the inclusion of only patients diagnosed with NTM-PD; and the exclusion of 21 patients with two or more positive SS/IS cultures who did not undergo BAL, as well as 13 patients who underwent BAL but not IS. Nevertheless, the patients underwent thorough study and evaluation using multiple diagnostic methods simultaneously.

## 5. Conclusions

We believe that sputum induction and fibrobronchoscopy are strongly recommended in the diagnostic workup of suspected NTM-PD to meet the microbiological criterion. They can be used sequentially or even together, depending on patient priorities and clinical context, to ensure the isolation and identification of mycobacteria in culture [2,3]. In patients with the nodular bronchiectasis form, BAL was essential for diagnosis in 58% of cases. For patients with the fibro-cavitary form of the disease, culture of two induced sputum samples collected on different days could offer a diagnostic efficacy comparable to BAL; however, we currently do not have sufficient data to validate this observation. Conversely, the high diagnostic success of BAL for NTM-PD in the nodular bronchiectasis form could allow us to shorten the diagnostic workup of these patients by limiting IS to a single sample and then proceeding directly with bronchoscopy and BAL.

## Figures and Tables

**Table 1:** Characteristics of patients according to the NTM species isolated.

NTM	Patients	M (%)	F (%)	Median Age (IQR)	Bronchiectatic Pattern	Fibrocavitary Pattern	New Cases vs. Recurrences	Immunosuppression	Comorbidities
*M. avium*	31 (32%)	6 (19%)	25 (81%)	72 (68–79)	22 (71%)	9 (29%)	27 vs. 4	3 (10%)	27 (87%)
*M. chimaera*	29 (30%)	11 (38%)	18 (62%)	67 (58.5–73)	22 (76%)	7 (24%)	19 vs. 10	5 (17%)	27 (93%)
*M. intracellulare*	21 (21%)	12 (57%)	9 (43%)	64 (60.5–69.5)	13 (62%)	8 (38%)	20 vs. 1	3 (14%)	18 (86%)
*M. abscessus*	5 (5%)	2 (40%)	3 (60%)	81 (64.5–83)	4 (80%)	1 (20%)	5 vs. 0	1 (20%)	5 (100%)
*M. kansasii*	5 (5%)	1 (20%)	4 (80%)	69 (64.5–75)	1 (20%)	4 (80%)	5 vs. 0	3 (60%)	5 (100%)
*M. xenopi*	4 (4%)	0	4 (100%)	51 (40–62)	2 (50%)	2 (50%)	4 vs. 0	1 (25%)	4 (100%)
*M. fortuitum*	2 (2%)	2 (100%)	0	65.5	1 (50%)	1 (50%)	2 vs. 0	1 (50%)	2 (100%)
*M. scrofulaceum*	1 (2%)	1 (100%)	0	67	0	1 (100%)	1 vs. 0	0	1 (100%)
Total	98 (100%)	35 (36%)	63 (64%)	68 (62–76.25)	65 (66%)	33 (34%)	83 (85%) vs. 15 (15%)	17 (17%)	89 (91%)

Abbreviations: M: males; F: females; NTM: nontuberculous mycobacteria; IQR: interquartile range.

**Table 2:** Characteristics of NTM patients according to the radiological appearance of lung lesions.

Patients	Total	M (%)	F (%)	Median Age (IQR)	New Cases (%)	Recurrences (%)	Immunosuppression (%)	Comorbidities (%)
Bronchiectatic pattern	65 (66%)	16 (25%)	49 (75%)	69 (77.5–63)	57 (88%)	8 (12%)	11 (17%)	58 (89%)
Fibrocavitary pattern	33 (34%)	19 (58%)	14 (42%)	68 (76–59)	26 (79%)	7 (21%)	6 (18%)	31 (94%)
Total	98 (100%)	35 (36%)	63 (64%)	68 (76.3–62)	83 (85%)	15 (15%)	17 (17%)	89 (91%)

Abbreviations: M: males; F: females; NTM: nontuberculous mycobacteria; IQR: interquartile range.

**Table 3:** Results of microscopy, PCR test for NTM, and cultures of IS and BAL according to the type of mycobacterium isolated.

NTM	Patients	IS Total	IS Smear Positive	IS Smear Negative	IS PCR Positive	IS PCR Negative	IS Culture Positive	IS Culture Negative	BAL Total	BAL Smear Positive	BAL Smear Negative	BAL PCR Positive	BAL PCR Negative	BAL Culture Positive	BAL Culture Negative	BAL Diagnostic/Patients
*M avium*	31 (32%)	48	6/45	39/45	15/43	28/43	19/48	29/48	31	15/24	9/24	15/18	3/18	31/31	0/31	15/31
*M chimaera*	29 (30%)	43	8/41	33/41	11/37	26/37	18/40	22/40	29	8/20	12/20	15/20	5/20	26/29	3/29	14/29
*M intracellulare*	21 (21%)	34	3/31	28/31	10/30	20/30	14/34	20/34	21	6/16	10/16	11/15	4/15	20/21	1/21	9/21
*M abscessus*	5 (5%)	10	3/10	7/10	4/10	6/10	6/10	4/10	5	2/3	1/3	2/3	1/3	4/5	1/5	3/5
*M kansasii*	5 (5%)	9	2/9	7/9	5/7	2/7	8/9	1/9	5	2/4	2/4	3/4	1/4	5/5	0/5	1/5
*M xenopi*	4 (4%)	8	0/8	8/8	0/8	8/8	0/8	8/8	4	2/4	2/4	0/2	2/2	4/4	0/4	4/4
*M fortuitum*	2 (2%)	3	1/3	2/3	1/3	2/3	1/3	2/3	2	1/2	1/2	1/1	0/1	2/2	0/2	1/2
*M scrofulaceum*	1 (2%)	1	1/1	0/1	1/1	0/1	1/1	0/1	1	1/1	0/1	1/1	0/1	1/1	0/1	0/1
Total	98 (100%)	156	24/148 (16%)	124/148	47/139 (34%)	92/139	67/153 (44%)	86/153	98	37/74 (50%)	37/74	48/64 (75%)	16/64	93/98 (95%)	5/98	47/98 (48%)

Abbreviations: NTM: nontuberculous mycobacteria; IS: induced sputum; BAL: bronchoalveolar lavage; PCR: polymerase chain reaction.

**Table 4:** Comparison of culture results in IS and BAL.

Radiologic Pattern	Sample	Positive Cultures for NTM	Negative Cultures for NTM	Total Cultures	Difference of Proportions, (95%CI), *p* Value
Nodular bronchiectatic	IS	30 (30%)	69 (70%)	99	<0.0001 (52.3224% to 73.7537%)
	BAL	62 (95%)	3 (5%)	65	
Fibrocavitary	IS	35 (65%)	19 (35%)	54	=0.0022 (11.3533% to 43.0198%)
	BAL	31 (94%)	2 (6%)	33	
Total	IS	67 (44%)	86 (56%)	153	<0.0001 (40.9085% to 59.1429%)
	BAL	93 (95%)	5 (5%)	98	

Abbreviations: NTM: nontuberculous mycobacteria; IS: induced sputum; BAL: bronchoalveolar lavage; CI: confidence interval.

**Table 5:** Microscopy results, PCR test for NTM, and IS and BAL cultures according to radiological classification.

Patients	Total	IS Total	IS PCR Positive	IS PCR Negative	IS Culture Positive	IS Culture Negative	BAL Total	BAL PCR Positive	BAL PCR Negative	BAL Culture Positive	BAL Culture Negative	BAL Diagnostic/Patients
Bronchiectatic pattern	65 (66%)	102	24/91 (26%)	67/91	30/99 (30%)	69/99	65	26/34 (76%)	8/34	62/65 (95%)	3/65	38/65 (58%)
Fibrocavitary pattern	33 (34%)	54	23/48 (48%)	25/48	35/54 (65%)	19/54	33	22/30 (73%)	8/30	31/33 (94%)	2/33	9/33 (27%)
Total	98 (100%)	156	47/139 (34%)	92/139	67/153 (44%)	86/153	98	48/64 (75%)	16/64	93/98 (95%)	5/98	47/98 (48%)

Abbreviations: PCR: polymerase chain reaction; IS: induced sputum; BAL: bronchoalveolar lavage.

**Table 6:** Comparison of PCR results in IS and BAL.

Radiologic Pattern	Sample	Positive PCR for NTM	Negative PCR for NTM	Total PCR	Difference of Proportions, (95%CI), *p* Value
Nodular bronchiectatic	IS	24 (26%)	67 (74%)	91	<0.0001 (30.7815% to 63.7288%)
	BAL	26 (76%)	8 (24%)	34	
Fibrocavitary	IS	23 (48%)	25 (52%)	48	=0.0308 (2.5126% to 43.4133%)
	BAL	22 (73%)	8 (27%)	30	
Total	IS	47 (34%)	92 (66%)	139	<0.0001 (26.6112% to 52.6084%)
	BAL	48 (75%)	16 (25%)	64	

Abbreviations: NTM: nontuberculous mycobacteria; IS: induced sputum; BAL: bronchoalveolar lavage; PCR: polymerase chain reaction; CI: confidence interval.

**Table 7:** Comparison of culture and PCR results in IS and BAL.

Sample	Method	% Positive	Sample Size n.	Difference of Proportions, (95%CI), *p* Value
IS	culture	44%	153	=0.0810 (−1.2037% to 20.8028%)
	PCR	34%	139	
BAL	culture	95%	98	=0.0002 (9.0504% to 32.1582%)
	PCR	75%	64	

Abbreviations: IS: induced sputum; BAL: bronchoalveolar lavage; PCR: polymerase chain reaction; CI: confidence interval.

## Data Availability

All relevant data are within the manuscript. Raw data are accessible, if requested, from the National Institute for Infectious Diseases “L. Spallanzani” Library to the following e-mail address: biblioteca@inmi.it.
